# Water, Sanitation, and Hygiene (WaSH) insecurity in unhoused communities of Los Angeles, California

**DOI:** 10.1186/s12939-023-01920-8

**Published:** 2023-06-01

**Authors:** Lourdes Johanna Avelar Portillo, Georgia L. Kayser, Charlene Ko, Angelica Vasquez, Jimena Gonzalez, Diego Jose Avelar, Nayib Alvarenga, Meredith Franklin, Yao-Yi Chiang

**Affiliations:** 1 Herbert Wertheim School of Public Health and Human Longevity Science, University of California (UCSD), 9500 Gilman Drive, La Jolla, CA 92093 USA; 2 Benioff Homelessness and Housing Initiative, School of Medicine, University of California San Francisco, 2789 25th Street, San Francisco, CA 94110 USA; 3 Spatial Sciences Institute, University of Southern California (USC), 3616 Trousdale Parkway, Los Angeles, CA 90089 USA; 4 Department of Statistical Sciences, University of Toronto, 700 University Ave., Toronto, ON Canada; 5 Department of Computer Science and Engineering, University of Minnesota, 200 Union St. SE, Minneapolis, MN 55455 USA

**Keywords:** WaSH, Homelessness, Unhoused communities, Los Angeles, Water insecurity, WaSH access, Access disparities, Urban populations

## Abstract

**Background:**

Access to water and sanitation is a basic human right; however, in many parts of the world, communities experience water, sanitation, and hygiene (WaSH) insecurity. While WaSH insecurity is prevalent in many low and middle-income countries, it is also a problem in high-income countries, like the United States, as is evident in vulnerable populations, including people experiencing homelessness. Limited knowledge exists about the coping strategies unhoused people use to access WaSH services. This study, therefore, examines WaSH access among unhoused communities in Los Angeles, California, a city with the second-highest count of unhoused people across the nation.

**Methods:**

We conducted a cross-sectional study using a snowball sampling technique with 263 unhoused people living in Skid Row, Los Angeles. We calculated frequencies and used multivariable models to describe (1) how unhoused communities cope and gain access to WaSH services in different places, and (2) what individual-level factors contribute to unhoused people’s ability to access WaSH services.

**Results:**

Our findings reveal that access to WaSH services for unhoused communities in Los Angeles is most difficult at night. Reduced access to overnight sanitation resulted in 19% of the sample population using buckets inside their tents and 28% openly defecating in public spaces. Bottled water and public taps are the primary drinking water source, but 6% of the sample reported obtaining water from fire hydrants, and 50% of the population stores water for night use. Unhoused people also had limited access to water and soap for hand hygiene throughout the day, with 17% of the sample relying on hand sanitizer to clean their hands. Shower and laundry access were among the most limited services available, and reduced people’s ability to maintain body hygiene practices and limited employment opportunities. Our regression models suggest that WaSH access is not homogenous among the unhoused. Community differences exist; the odds of having difficulty accessing sanitation services is two times greater for those living outside of Skid Row (Adj OR: 2.52; 95% CI: 1.08–6.37) and three times greater for people who have been unhoused for more than six years compared to people who have been unhoused for less than a year (Adj OR: 3.26; 95% CI: 1.36–8.07).

**Conclusion:**

Overall, this study suggests a need for more permanent, 24-h access to WaSH services for unhoused communities living in Skid Row, including toilets, drinking water, water and soap for hand hygiene, showers, and laundry services.

**Supplementary Information:**

The online version contains supplementary material available at 10.1186/s12939-023-01920-8.

## Background

Access to water and sanitation are recognized as a basic human right. The United Nations (UN) General Comment 15 on the right to water, for instance, issued by the Committee on Economic, Social and Cultural Rights declares that a person has the right to have sufficient, safe, physically accessible, and affordable water for personal needs without any form of discrimination [1]. Additionally, in 2010, through the Resolution 64/292, the UN’s General Assembly recognized the human right to water and sanitation [2]. The inclusion of water and sanitation as a human right may seem like an advancement. However, the most recent global water report estimates that 2 billion people worldwide lack safely managed drinking water and 3.6 billion lack safely managed sanitation services [3]. Additionally, access to hygiene remains an unrecognized human right globally, thus, further limiting progress in improving public health. There are different definitions used for water and or sanitation insecurity [4–8], and little distinction in addressing drinking water, sanitation, and hygiene (WaSH) insecurity. In this study, we define WaSH insecurity as the absence of basic WaSH services as defined by the Joint Monitoring Programme (JMP). This definition also incorporates the relational inequities in WaSH access as defined by Adams et al. (2021) resulting in WaSH insecurity experiences that increases an individual’s exposure to preventable health risks [3, 9]. Experiences of WaSH insecurity are disproportionally found among impoverished communities living in low and middle-income countries (as seen in the work of [7, 10, 11]). However, WaSH insecurity is also present in high-income countries like the United States (US), where vulnerable communities, including unhoused people, lack continuous access to safe, sufficient, reliable, and affordable WaSH services. Unfortunately, little is known of the true magnitude of WaSH insecurity experienced among unhoused people in the US, as the needs of unhoused people remain heavily underexplored, leaving these communities underserved.

While the term “homeless” continues to dominate mainstream discourse and government reports, it stigmatizes people with lived experience. Therefore, throughout this paper, we use the term “unhoused” to refer to people or individuals with lived experience of sheltered or unsheltered homelessness. The term unhoused is preferred and used by grassroots organizations and people with lived experience in the community because it maintains the humanity of the people discussed [12].

Addressing the WaSH service needs of unhoused communities is critical. In global water reports, the US often claims to have universal access to safely managed drinking water and sanitation services [5, 13, 14]. The JMP of the World Health Organization (WHO) and the United Nations Children's Fund (UNICEF), for example, produce global estimates on progress made related to WaSH. In JMP's most recent assessment based on 2020 household data, the US reported that 97% of its housed urban population had access to safely managed drinking water from acceptable sources that are free of contaminants [3]. The US also reported that roughly 98% of its housed urban population had access to safely managed sanitation services, with no data provided for hygiene services [3]. However, the literature on household WaSH insecurity suggests it does exist in the US and it disproportionally affects migrant farming communities, Indigenous communities, and low-income urban communities [5, 6, 15–18]. Reports such as the ones provided by the JMP are limited by the data countries share. In the US, water estimates come from the American Housing Survey (AHS) and the US Environmental Protection Agency's Safe Drinking Water Information System datasets [14, 19–21]. The unit of measurement on which these datasets are based is at the household unit, which automatically excludes unhoused people. While initiatives are underway to count unhoused communities in census data, there are no up to date datasets available that accounts for the WaSH needs and WaSH insecurity experiences of unhoused communities in the US.

Restricting WaSH insecurity to household-level analyses excludes the lived experiences and service needs of those who do not have access to permanent housing. While limited, the WaSH insecurity literature that focuses on unhoused communities suggests that poor access to services leads to and perpetuates a cycle of poverty [22, 23]. In other words, for unhoused people, WaSH insecurity exacerbates their stigmatization and social exclusion. For example, DeMyers, Warpinski, and Wutich’s (2017) study in Phoenix, Arizona, found that based on people's living conditions (in shelters, encampments, and with or without a roof), WaSH insecurity affects people differently. At the same time, WaSH insecurity can prevent the transition out of homelessness by aggravating health problems that contribute to mental and physical health deterioration and joblessness, all of which may increase a person’s susceptibility to long-term homelessness [22]. Similarly, Leibler et a. (2017) study in Boston, Massachusetts, found that poor access to hygiene facilities (and consequently, poor hygiene practices) led to poor physical health and increased risk of infectious diseases, evident in unhoused people who cope with mental health problems and substance use [24]. In Fresno, California, Speer (2016) found that the lack of infrastructural WaSH access in cities is an example of the aggressive policies aimed to criminalize, exclude, and remove encampments from public spaces [25]. This limited access to WaSH infrastructures, including sanitation facilities forces unhoused people to practice open defecation, as seen in Capone et al.'s (2018) study in Atlanta, Georgia. Capone and colleagues found thirty-nine open defecation sites near shelter and soup kitchens that tested positive for pathogens, which poses an increased risk of infection by faecal-oral route in unhoused communities [19, 26]. Furthermore, the criminalization of unhoused communities pushes people into hazardous spaces and further disconnects them from much-needed services [27, 28]. Pushing unhoused people into hazardous environments is seen in the work of Flanigan and Welsh (2020) that found unhoused people living along the San Diego River were more socially isolated and disconnected from services compared to those living in downtown areas [28]. Flanigan and Welsh report that unhoused people lived along the riverbed to avoid police harassment and encampment sweeps [28]. In addition to creating barriers to access safe WaSH services, living in secluded areas raises the risk of exposure to contaminated water and disease outbreaks [28–30].

Previous work on WaSH access among the unhoused has been limited in scope, and three main research gaps exist. First, from a geographic perspective, research in Los Angeles, an area with the second-highest count of people experiencing homelessness across the nation, roughly 66,436 people, on a single night in January 2020, remains underexplored [31]. To the authors ' knowledge, no studies have addressed the WaSH insecurity experiences and WaSH service needs of unhoused people living in Los Angeles (with the exception of local efforts that report on the lack of public sanitation facilities, trash receptacles, and public fountains [32–34]). Second, no known study has comprehensively assessed WaSH insecurity among the unhoused in Los Angeles, including drinking water, sanitation, and hygiene (showers, laundry services, and handwashing stations). Understanding the interim-level services, specifically the WaSH service needs of unhoused communities, is crucial for informing policy and creating programs that address and improve people’s health outcomes and living environments. Lastly, the temporal aspects of WaSH insecurity have not been fully recognized or considered. Given that the hours of operation can vary for any given service, limits to WaSH access can force the unhoused to resort in unsafe WaSH coping strategies. With the exception of Kuhlmann et al.’s (2019) study on menstrual hygiene access in Missouri [35] and the study by Maroko et al. (2021) on sanitation access in New York [36], no known study has captured how WaSH access changes throughout the day for unhoused communities.

In this study, we explored WaSH insecurity in Los Angeles, a city that has historically been struggling with a homelessness crisis [37, 38] with the goal of shedding light on the WaSH insecurity experiences that unhoused communities face. We focus our research in the community of Skid Row, a 50 block area in Downtown Los Angeles where an estimated 4,662 people experience homelessness in a single night, and roughly 2,100 live outdoors in tents, vehicles, and makeshift shelters [39]. While the community of Skid Row hosts one of the largest encampments, it is also confronted with public health equity issues raised by both community-based efforts [32–34] and the County of Los Angeles Department of Public Health [40]. The Los Angeles Community Action Network (LA CAN) released two reports in 2013 and 2017 that advocate for the City of Los Angeles to improve the access to water, sanitation, and consistent trash collection [33, 34]. In 2017, the Los Angeles Central Providers Collaborative (LACPC), a community-based group of Skid Row residents and grassroots organizations, released an audit report of public toilets available in the community. The report found that only nine public toilets were available in Skid Row for a population of roughly 1,777 unsheltered individuals in 2017 [32]. This is approximately 198 unhoused people per toilet. Thus, our study is significant because it will contribute to existing local knowledge and help to expand our understanding of WaSH insecurity experienced by unhoused communities and the daily barriers unhoused people encounter in accessing services. The knowledge and findings gathered from this study will also bring new insights into the persistent inequities of WaSH access experienced by unhoused communities.

This study moves beyond the household to study WaSH insecurity in Los Angeles, California, to address the unmet needs of unhoused communities. Specifically, this research addresses two main questions: (1) How do unhoused communities cope and gain access to WaSH services in different places? and (2) What individual level factors (gender identity, racial/ethnic, age, sleeping location, and duration a person has been unhoused) contribute to unhoused people’s ability to access WaSH services? Ultimately, this study seeks to advance our understanding of WaSH insecurity for unhoused people in Los Angeles to highlight the need for interim-level services that can help improve people's lives and health through affordable, safe, and reliable access to WaSH services.

## Methods

This is an observational cross-sectional study of (*N* = 263) unhoused people with lived experience in the Los Angeles area. All study participants reported living in the Los Angeles region at the time of the interview. The surveys were collected in both Spanish and English in two months (June and July) in the summer of 2019. Before data collection, all study activities were reviewed and approved by the University of Southern California Institutional Review Board (IRB) (Protocol UP-18–00323). Participation in the study was anonymous and voluntary, and only unhoused adult participants over 18 who gave oral consent were enrolled. This study defines an unhoused person as those experiencing unsheltered homelessness (e.g., living in a public or private place not designated for sleeping, in the streets, in tents, vehicles, or other forms of makeshift housing). At the same time, those people experiencing sheltered homelessness, such as people living in emergency shelters, transitional housing programs, motels, hotels, or safe havens also formed part of this definition as they do not have a permanent and stable place to live [41].

**Fig. 1:**
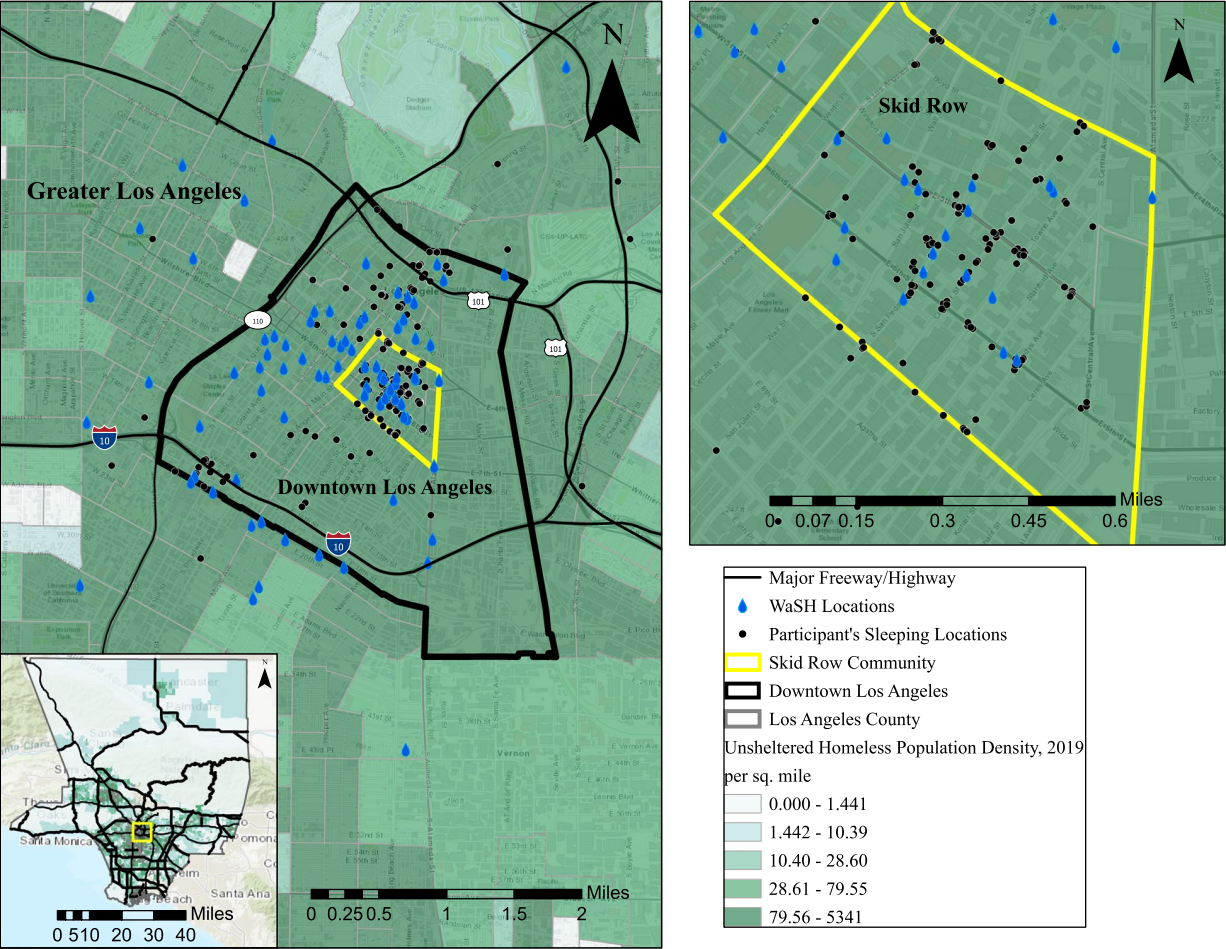
Study area and study participants overlapped with LA County’s unhoused population density. Source: Los Angeles Homeless Services Authority point-in-time estimated in 2019

Only study participants who consented to and completed the questionnaire were included in the final analysis. Participants were gifted a meal card, bottled water, and hygiene kits regardless of whether they fully completed the survey interview process. The study mainly focused on the community of Skid Row (Fig. 1). However, while interviewed within the Skid Row community boundaries, some participants reported sleeping in other areas, including downtown Los Angeles and greater Los Angeles neighbourhoods, as seen in Fig. 1. To protect the privacy and confidentiality of study participants, the sleeping locations shown in Fig. 1 are not the exact locations but placed at random within the street segment boundaries they reported frequently sleeping. Overall, our decision to focus on Skid Row was both pragmatic and strategic. Annual street counts conducted by Los Angeles Homeless Services Authority (LAHSA) routinely find many more unhoused communities in Skid Row than in the other neighbourhoods of Los Angeles. Furthermore, this is an area that historically has been a containment zone where most of the services and encampments are located [38].

### Target population

This study used a mixture of convenience and snowball sampling strategies to recruit participants, as participants are difficult to reach. Participants included unhoused participants who resided within the Skid Row community boundaries, stretching from 3rd to 7th Street (North and South) and Alameda to Main Street (East and West). Additionally, passers-by within the designated study area, which on occasion extended to participants in nearby tents and friends of participants, formed part of the study. The sample population also included LavaMae^x^ © non-profit organization guests in two service locations of downtown Los Angeles: City Hall and St. Francis Center. This partnership with LavaMae^x^ helped provide the research team with a safe space to conduct surveys. In exchange, the team provided hygiene kits and bottled water to guests and people in nearby encampments, regardless of study participation.

### Survey data and data analysis

The survey instrument consisted of semi-structured and open-ended questions that explore the WaSH access and coping strategies of people with lived experience of homelessness. The survey was first piloted among the research team to improve the quality of questions. Then, the survey instrument was tested in Skid Row with thirty unhoused participants before making final revisions. Each survey took approximately 30–90 min to complete. The survey instrument asked a series of demographic questions, including different living conditions, areas where they often rest at night, WaSH accessibility, and general health information. WaSH access questions were collected to represent different types of WaSH services utilized at different times of the day (e.g., morning, afternoon, and night).

**Fig. 2:**
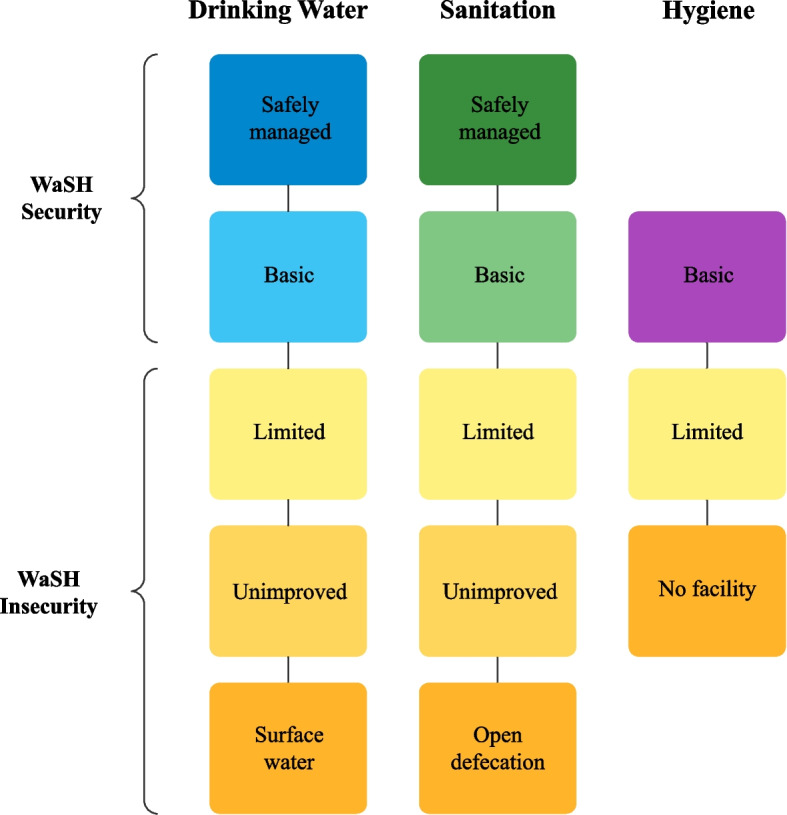
Drinking water, sanitation, and hygiene (in)security categorization. Note: Categorizations were adapted from Alhelí Calderón-Villarreal and the Joint Monitoring Programme, 2022. Source: https://washdata.org/monitoring

In our study, we measured WaSH insecurity as those people who lack access to basic and or safely managed WaSH facilities based on the JMP definitions and service ladder (see Fig. 2). According to the JMP, access to drinking water is based on a five-step service ladder with safely managed drinking water access at the top, which considers whether the service is accessible on premises, available at all times, and free from contaminants [42]. Basic drinking water access refers to the use of improved water sources located within 30-min roundtrip. Improved drinking water sources are those that provide accessible, continuous, and safe water, including those from piped water systems, boreholes, protected wells and springs, packaged water, delivered water, and rainwater [42].Limited access refers to drinking water from an improved source that exceeds 30-min roundtrip to collect. Unimproved water access is drinking water from an unprotected well or spring. At the bottom of the service ladder is surface water access that refers to drinking water directly from a stream, river, canal, lake, pond, and dam [42].

Sanitation is defined as the ability to safeguard access to facilities that are not shared among other households and utilizing improved sanitation sources designed to hygienically separate excreta from human contact. Meaning that human excreta is treated and disposed of in situ, stored temporarily and then treated off-site, or transported through a sewer system with wastewater and then treated off-site [43]. Access to sanitation is also based on a five-step ladder that includes: at the top safely managed sanitation which refers to the use of improved sanitation facilities that are not shared with other households and where excreta are safely disposed of in situ or removed and treated offsite. If people use improved facilities that are not shared with other households but where excreta are not safely managed, then people using those facilities are classed as having a basic sanitation access. In the middle of the latter is limited sanitation access which is the use of improved facilities that are shared between households. Unimproved sanitation is the use of pit latrines without a slab, hanging latrines, or buckets. Lastly, at the bottom of the ladder is open defecation which is the disposal of human feces in open fields, open bodies of water, bushes, and other open spaces or with solid waste [43]. For both drinking water and sanitation access, our study expands the analysis to integrate continuity, an important component (as stated in [8]) given that our study population may not have access to these services 24-h, which are not incorporated in the JMP monitoring reports.

Furthermore, the JMP defines hygiene as having the ability to practice handwashing and safely manage menstruation [44]. According to the JMP, “hygiene refers to the conditions and practices that help maintain health and prevent spread of disease including handwashing, food hygiene, and menstrual hygiene management” [44]. This definition is based on a three-step ladder that includes, having basic availability of soap and water at home at the top of the ladder, having limited access to soap or water at home, and having no facility available on premises, at the bottom of the ladder [44]. Since participants in this study are unhoused, these categorizations do not adequately apply. Therefore, this study expands on hygiene access outside the household to the sleeping areas of participants where they practice daily hygiene practices, including showering, handwashing, clothing change, and laundering. The JMP typically measures hygiene in terms of hand hygiene practices, menstruation, and food hygiene. However, in this study, we expand the analysis to incorporate body hygiene that addresses unhoused communities' laundry and shower needs. Adding these two variables is important because current literature indicates that poor access to hygiene practices impacts people's self-esteem and the way others view them in their communities, which limits their ability to seek out services and employment [22, 23, 36, 45].

The survey also collected health variables. Participants were asked whether they have had any health problems within 30 days from the interview date to understand better the health risks of people who are unhoused and possibly exacerbated by the inadequate access to WaSH services. Lastly, participants were provided with a space to express their main concerns and overall experiences in navigating access while being unhoused.

The data was collected using paper surveys, and each survey had a unique study identification. Each paper survey was abstracted and coded onto a database. In total, we collected 280 surveys, in which 17 were incomplete surveys that did not form part of the final analysis. Each variable coded was verified using a survey metadata. After completing the data abstraction and coding, it was reviewed and verified twice before entering the analysis phase. The coded data were imported into R Studio version 1.3.1093 to conduct statistical analyses. Participants' sleeping locations at the time of their interview were geocoded using Esri Survey123, matched to the survey data, and then visualized using ArcGIS Pro version 2.7.

To address the first research question, we examined individual data to summarize the coping strategies and types of WaSH services the sampled population reported accessing using descriptive statistics. To measure our second research question focusing on whether differences exist among unhoused communities, we utilized individual-level factors that may lead to difficulty accessing WaSH services. Specifically, we integrated generalized linear models (GLM) to measure the association between difficulty accessing different WaSH services and individual-level characteristics. The reasoning for choosing GLM as opposed to other statistical models is that it does not assume the dependent variable to be normally distributed. Additionally, the outcome variable in the models, “difficulty accessing toilets, showers, laundry, drinking water, or handwashing stations,” is binary (Yes/No). The independent categorical variables in the models included: gender identity, race and ethnicity, age, sleeping location, and duration a person has been unhoused. Associations tested were chosen based on reviewing relevant literature and observing the lack of studies exploring heterogeneity among unhoused groups as it pertains to inequities in WaSH access.

## Results

### Population characteristics

A total of 263 participants were included in the final analysis of this study. The housing status of participants varied across the sample: 25 stayed in emergency shelter systems, three in transitional housing programs, four stayed with family, and the remaining 231 participants reported sleeping in different unsheltered conditions at the time of interview. In this study, unsheltered living conditions refer to people sleeping in tents, makeshifts, vehicles, freeway bypasses, and other conditions without a roof. Table 1 summarizes our study population demographics. Half of the study participants enrolled (*n* = 134) reported sleeping within the Skid Row community boundaries (7th and 3rd and Alameda and Main streets). However, some participants also reported sleeping in other communities outside of Skid Row boundaries, including downtown Los Angeles (*n* = 92) and the greater Los Angeles area encompassing Santa Monica, Venice, Hollywood, and South Los Angeles areas (*n* = 37). Seventy percent of the population identified as male. The mean age was forty-eight years old. Only eight participants younger than 24 years enrolled in the study. Black or African American (41%) and Latinx (30%) people overrepresented the sampled population. People identifying as White were 15% of the study sample population. In total, 84% of the study population have been continuously unhoused for more than a year. The sampled population reported experiencing homelessness for sixty-five months (5.4 years) on average, with only 41 participants reporting being unstably housed for less than a year.

**Table 1:** Frequency distribution of sample population demographics (*N* = 263)

Characteristic	Category	Count (%)
Housing status	Unsheltered	231 (87.83)
	Emergency shelters	25 (9.51)
	Family/friends	4 (1.52)
	Transitional housing	3 (1.14)
Community	Skid Row	134 (50.95)
	Downtown Los Angeles	92 (34.98)
	Greater Los Angeles	37 (14.07)
Gender identity	Male	180 (68.44)
	Transgender male	2 (0.76)
	Female	79 (30.04)
	Transgender female	1 (0.38)
	Missing	1 (0.38)
Age	18 to 24	8 (3.04)
	25 to 34	33 (12.55)
	35 to 44	55 (20.91)
	45 to 54	69 (26.24)
	55 to 61	60 (22.81)
	Greater than or equal to 62	32 (12.17)
	Missing	6 (2.28)
Race/ethnicity	Black/African American	109 (41.45)
	Latinx/Hispanic	80 (30.42)
	White	39 (14.83)
	Another group	17 (6.46)
	American Indian/Alaskan Native	11 (4.18)
	Asian and Pacific Islander	3 (1.14)
	Missing	4 (1.52)
Sexual orientation	Heterosexual	222 (84.03)
	Bisexual	18 (6.34)
	Homosexual	13 (4.94)
	Asexual	1 (0.38)
	Other	2 (0.76)
	Missing	8 (3.04)
Duration of homelessness	Less than 1 year	41 (15.59)
	1–3 years	104 (39.54)
	4–6 years	42 (15.97)
	7 years or greater	65 (24.71)
	Missing	11 (4.18)

Percentages equal to totals within each demographic characteristic

To understand participants' current housing status, we inquired about the contributing factors that led participants to become unhoused. Table 2 highlight these factors, with 27% of participants reporting unemployment being the cause of why they are unhoused. Sixteen percent of participants reported that their loss of housing resulted from the lack of affordable housing in Los Angeles and their inability to pay rent resulting in eviction. Substance use and misuse was the third most reported cause of a person being unhoused (15%), followed by family conflict (15%). Furthermore, roughly eight percent of the sample population reported the cause for being unhoused was due to their immigration status or criminal record. Lastly, a small percentage (3%) of participants reported being unhoused due to being victims of domestic violence.

**Table 2:** Frequency distribution of variables associated with causes of homelessness

Cause	Count (%)
Unemployment	95 (26.46)
Unaffordable housing/eviction	57 (15.88)
Drug/alcohol misuse	55 (15.32)
Family conflict	54 (15.04)
Other^a^	30 (8.36)
Mental health disorder	26 (7.24)
Family/spousal death	16 (4.46)
Physical disability	15 (4.18)
Domestic violence/sexual abuse	11 (3.06)

The percentages are based on 359 responses given by *N* = 263 since this was a multiple-response question

^a^The “other” category includes immigration status and formerly incarcerated people/recently released from prison

Participants were also asked about the number of times they were forced to move their tents because of encampment sweeps enforced in different parts of the city by law enforcement. On average, the sample population reported moving their tents and belongings at least nine times within a 30-day period. Forty-one participants reported moving every day due to encampment sweeps. Ninety-nine participants (38%) also reported being cited for misdemeanors including not moving their tents, public urination, or jaywalking within 30-days from time of interview.

### Drinking water access

In this study, zero percent of the sampled population reported safely managed drinking water available on household premises given that people are unhoused. Seventy-one percent of the sample population reported having at least basic access to improved drinking water from sources within 30 min roundtrip of where they slept in the morning time. In other words, they obtained water from improved sources such as purchased bottled water, asked business establishments for free water, or refilled plastic bottles using public fountains found in parks and libraries. Ten percent of the sample population reported limited access to drinking water sources in the morning, which refers to people walking more than 30-min roundtrip to obtain water. The remaining 13% reported varying distances to obtain drinking water in the morning. Lastly, six percent of the sample population reported illegally opening fire hydrants to meet their daily drinking water needs in the morning. Access to drinking water, however, shifted at nighttime. While 81% of the sample reported having basic access to drinking water, roughly 50% of these participants refilled plastic bottles or purchased water during the day and stored it for night use. Access is reduced in the evening due to limited hours of operations in the main places people use to obtain water, including supermarkets, dollar stores, and public facilities (parks and libraries) that are not open overnight. At the same time, participants expressed safety concerns walking a few blocks at night to obtain drinking water from the few non-profits open at night. Only three percent of the sample population reported limited access to water and walking more than 30-min to obtain water at night, and six percent continued to rely on using fire hydrants for drinking water. When asked about the total water intake in a day, more than half of the sampled population (54%) reported an intake of up to three (16 oz) bottles of water a day, 31% reported drinking up to six bottles, and only 13% reported drinking more than six bottles of water per day (2% of the sample did not provide a response).

### Sanitation access

**Fig. 3:**
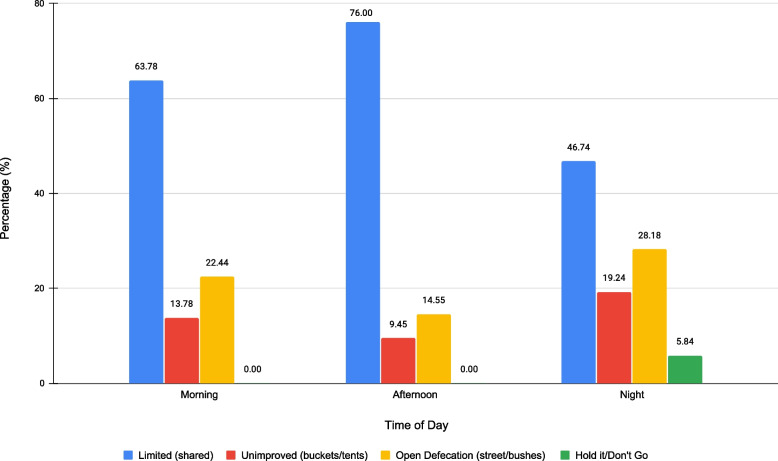
Access to different sanitation facilities based on time of the day (*n* = 263). *Note*: The categorization is based on the Joint Monitoring Programme benchmark ladder for sanitation access

In addition to examining drinking water accessibility, participants reported their access to sanitation services. Based on the JMP’s sanitation access categorization, most of our study participants mainly reported having limited access to sanitation facilities, that is access to shared sanitation facilities that fluctuated throughout the day. Figure 3 illustrates the different types of access the sampled population reported based on time of day.

Throughout the day, participants reported heavy reliance on public facilities, including toilets in parks and libraries, staffed Pit Stop program toilets, and non-profit hygiene centers. Limited access to shared sanitation ranged from 63% in the morning, 76% in the afternoon, and 47% at night. At nighttime, limited access to sanitation facilities is further reduced, as only a few facilities are open and available overnight. The majority of the non-profit organizations and business establishments that people heavily rely on are not open overnight, with the exception of the ReFresh Spot, a community-driven project that provides free sanitation, shower, and laundry access available 24-h; and the People Concern hygiene center that provides free showers and laundry access. Both of these services are located in the community of Skid Row. As a result of reduced access to publicly available toilets at night, 19% of participants reported coping with unimproved sanitation (using buckets inside their tents or plastic bottles) and 28% reported openly defecating and urinating in public spaces. The lack of overnight sanitation facilities also resulted in other coping strategies; six percent of the sample population reported holding it at night and waiting until the morning to use a toilet due to inaccessibility and lack of safe sanitation. Overall, 58% of participants reported that while finding a toilet to urinate is challenging, it is easier to cope with compared to when finding a toilet to defecate.

### Hygiene

The JMP defines access to hygiene as the conditions and practices of handwashing, food hygiene, and menstrual hygiene management that help sustain good health and reduce the spread of disease [44]. As previously stated, these measures fall short of capturing body hygiene, such as having the ability to shower and wash clothes, all of which are necessary to maintain health and prevent the risk of infectious diseases. Therefore, hygiene access in this study encompasses handwashing, menstrual hygiene, body hygiene, and access to laundry services. In our study, zero percent of participants had handwashing stations with soap and water access where they slept. Participants reported using public facilities, including public toilets, business establishments, and non-profit organizations, to practice hand hygiene. Twenty eight percent of participants use these facilities to also refill plastic bottles with water that they use to rinse their hands inside their tents. Among the sampled population, 32% reported washing their hands with water, soap, and hand sanitizer before meals and after using sanitation facilities. Thirty percent of participants reported only having access to water and soap, while 17% reported only using hand sanitizer to clean their hands before meals and after using a toilet.

In terms of menstrual hygiene management, out of the 70 female participants interviewed in the sample population, fewer than half (*n* = 35) continue to have their menstrual cycle. Eight of these participants who still menstruate reported difficulty managing menstrual hygiene due to limited access to menstrual products (tampons and pads) and facilities where they can clean their clothing items and bathe. As such, women reported coping with using toilet paper or clothing items to manage menstruation. Furthermore, one woman reported that managing her cramps was a challenge while experiencing homelessness. Three of the unhoused women who still menstruate also reported feeling “dirty” and “smelly” due to not being able to afford and find menstrual products and access to showers. It is also important to note that six women in their reproductive years, were not taking any contraception, and reported no longer having their menstrual cycle. Unhoused women no longer experiencing their menstrual cycle may be attributed to trauma and stress from living on the streets.

Accessibility to shower facilities to maintain body hygiene practices was also limited among the unhoused participants in this study. Seventy-three percent of the participants reported showering less than three times per week. Overall, only 27% of the sampled population reported showering almost every day (4 days or more in the week). The most common bathing source reported (76%) were non-profit organizations, including the ReFresh Spot, shelters, LavaMae^x^, and the People Concern. In other instances, people used sinks in public parks and businesses to do a quick rinse with paper towels and water, also referred to by participants as a “bird bath” (13%). Participants also reported using buckets inside their tents to shower (6%). Participants reported that excessive wait times, an extensive waitlist system to access bathing facilities, or facilities being out of order further reduced their access to a shower. As a result, some participants reported coping by illegally tapping into fire hydrants to obtain water for bathing (*n* = 3) and using the Los Angeles River (*n* = 2).

Access to laundry services to maintain personal hygiene is also limited in unhoused communities. Across the sampled population, 91% of the participants reported washing their clothing items less than three times per month. Inaccessibility of laundry services was one of the most reported complaints. Study participants cited a lack of laundry facilities and being unable to afford paying for these services in private laundromats. There were only a few available laundry facilities dedicated to serving unhoused communities at the time this study took place. These included the ReFresh Spot that offers free laundry services, the People Concern that offers laundry services for a small fee, the Downtown Women's Center that offers free laundry service to women only, and the Laundry Truck LA mobile service that offers free services for a limited number of people per day. A small number of participants (a combined 14%) reported using buckets inside their tents or sinks from public parks to wash their clothes when they cannot access services provided by non-profits. Another 12% of participants reported it is easier to throw away their clothes than wash them due to reduced access to these services in the communities where they reside. For people sleeping in neighborhoods outside of Skid Row, specifically those residing in greater Los Angeles area (*n* = 37) during the daytime reported commuting to Skid Row, the area with the most services, to access different hygiene services. The commute to Skid Row from the places people sleep at night can range from between thirty minutes to one-hour one-way commute when traveling using public transportation and by foot.

### Health risks related to WaSH insecurity

In addition to collecting WaSH accessibility information, this study also collected health variables to capture health risks that may be associated with poor access to WaSH services. Table 3 represents a list of reported health outcomes that participants reported coping with within a 30-day period from the time of interview. The most common health problem reported was skin infections (39%) among the sampled population that can result from lack of access to adequate bathing facilities. Participants also reported experiencing migraine headaches (35%) and dehydration (34%) which can result from lack of sufficient water intake. At least ten percent of participants reported dealing with urinary tract infections within a 30-day period that may be related to limited access to drinking water, toilets, and voluntary urinary retention when there is no access to sanitation facilities at all times of the day. Diarrhea was also a commonly reported health condition that people were coping with around thirty days from the time of the interview (*n* = 55).

**Table 3:** Frequency distribution of health reported outcomes over a 30-day period

Category	Health Outcome	Count (%)
Hygiene related	Skin infection	64 (39.02)
	Diarrhea	55 (33.54)
	Fungus	27 (16.46)
	Head and body lice	9 (5.49)
	Typhus	5 (3.05)
	Hepatitis A	4 (2.44)
WaSH related	Migraine/headache	105 (35.12)
	Dehydration	103 (34.45)
	Constipation	59 (19.73)
	Urinary tract infection	32 (10.70)

Total sample is *N* = 263; however, this was a multiple-response question. As a result, counts do not sum up the total sample size. Percentages are based on people who indicated a health condition experienced in the past 30-days from the interview time. These percentages are aggregated per sub-section. Missing data (*n* = 8) did not form part of the calculation

### WaSH access inequities

We examined the individual-level factors that lead to difficulty accessing sanitation and hygiene services for the unhoused. Table 4 summarizes two of the model results from the generalized linear models. The models tested the factors associated with difficulty accessing sanitation (toilets) and handwashing facilities among the sampled population. The reference groups in each of these models included: White male, people between 19–38 years of age, duration time a person has been unhoused to be less than a year, and the Skid Row community. The results from the model indicate that when we compared all three communities where people reported sleeping, the odds of having difficulty accessing sanitation services are two times greater for those living in greater Los Angeles area than for those people living on Skid Row and downtown Los Angeles (Adj OR = 2.52, 95% CI: 1.08–6.37). Additionally, for people experiencing homelessness for more than six years, the odds of encountering difficulty accessing sanitation services were three times greater than those who experience homelessness for less than a year (Adj OR = 3.26, 95% CI: 1.36–8.07). Regarding racial differences in access to sanitation services, for people who identified as Black/African American and Latinx, the odds were 0.35 (95% CI: 0.13–0.84) and 0.30 (95% CI: 0.11–0.74), respectively, lower compared to unhoused people who identified as White, a minority group in the unhoused population residing in Los Angeles. Table 4 also illustrates the factors associated with difficulty accessing hand hygiene facilities. We found that unhoused people residing in greater LA area were almost three times more likely to report difficulty accessing handwashing facilities compared to those living in Skid Row and downtown (Adj OR = 2.53, 95% CI: 1.11–5.93).

**Table 4:** Generalized linear model (GLM) output results for difficulty accessing sanitation and hygiene facilities

	Sanitation			Handwashing		
Variable	Adj OR	Lower bound (95% CI)	Upper bound (95% CI)	Adj OR	Lower bound (95% CI)	Upper bound (95% CI)
Intercept	1.36	.39	4.89	.42	.12	1.42
Female	.98	.53	1.82	.93	.50	1.70
Race/Ethnicity						
White	-	-	-	-	-	-
Black/African American	.35*	.13	.84	1.05	.46	2.40
Latinx/Hispanic	.30**	.11	.74	.62	.27	1.46
Other	.49	.15	1.55	.77	.26	2.26
Age						
19–38	-	-	-	-	-	-
39–50	1.13	.46	2.79	2.41	.99	6.08
51–57	1.73	.72	4.18	1.63	.68	4.02
> 57	.90	.39	2.05	1.58	.70	3.71
Time (years)						
Less than a year	-	-	-	-	-	-
1–3	1.49	.66	3.41	.95	.42	2.15
4–6	1.66	.64	4.38	1.14	.44	2.96
More than six years	3.26**	1.36	8.07	1.03	.44	2.45
Community						
Skid Row	-	-	-	-	-	-
Downtown LA	.95	.51	1.76	1.36	.74	2.52
Greater LA	2.52*	1.08	6.37	2.53*	1.11	5.93

*Adj OR* Adjusted Odds ratio, *CI* Confidence interval

^*^p < 0.05

^**^p < 0.01

While no other statistically significant differences were observed for other WaSH services, including drinking water, shower, and laundry access, our sample population did report discrimination when accessing WaSH services. Thirty-nine percent of participants who identified as Black or African American and 42% of Latinx unhoused participants reported experiencing racial discrimination when trying to access WaSH services. Forty-two percent of participants that reported discrimination when accessing sanitation services were Black or African American and 26% were Latinx. Most reported places participants experienced discrimination when trying to access sanitation facilities was in business establishments (73%) and at public toilets (15%). Two of the main reasons participants reported being discriminated against when accessing sanitation facilities was due to the need to be a paying customer (45%) and appearance (29%). Additionally, 61% of the unhoused males in the sample reported experiencing gender discrimination more often when trying to access shower facilities compared to only 38% of unhoused women. Forty-three percent of these participants that experienced discrimination at shower facilities reported it took place in shelter systems. Furthermore, 46% of Black unhoused participants also reported discrimination when accessing laundry facilities. The most reported reason participants reported discrimination when accessing laundry facilities was due to appearance (45%) and inability to afford laundry services (15%).

## Discussion

WaSH insecurity impacts the lives of communities across the globe, including vulnerable unhoused communities in Skid Row, Los Angeles. Safe, equitable, sufficient, reliable, affordable, and dignified access to WaSH services is often not possible for unhoused people, especially at night. In Los Angeles, many of the unhoused participants we interviewed could not access sanitation at night, and shower or wash their clothes regularly. Our study findings suggest that unhoused communities embark on different survival coping strategies to access and meet their daily WaSH needs. Access to drinking water was reduced at nighttime which forces people to engage in purchasing and storing water during the day to sustain their needs. The majority of the sampled population reported having limited sanitation access throughout the day, but at night, people reported a higher percentage of public urination or defecation in buckets or plastic bottles inside their tents. Many also lack sufficient water for basic hand hygiene, showering, and laundry services. The lack of basic WaSH services also makes it difficult for unhoused women to manage their menstrual health hygiene safely. Overall, WaSH insecurity creates barriers for people to fully manage their health, seek employment, and improve their living conditions.

In Los Angeles, unhoused people live in an environment that is scarce of essential WaSH services, which further degrades their physical and mental health and reduces their opportunities for employment. In our study, the aspect of appearance was a common factor reported by participants in being discriminated in places, thus reducing their ability to access essential WaSH services. Meaning that WaSH insecurity serves both as a “driver and an inhibitor” of prolonged homelessness [22]. In our study, participants also reported difficulty accessing sanitation and hygiene services that would allow them to practice daily body hygiene that in essence would help them maintain an appearance of someone who is not homeless to be accepted in public spaces and be less discriminated when accessing other supportive services.

Menstrual hygiene management is an added challenge for unhoused women. In our study, the limited access to sanitation and hygiene facilities was a problem for the s population, especially for women managing their menstrual hygiene who reported feeling smelly and dirty due to the lack of shower facilities. Similarly, menstrual hygiene challenges among unhoused women were also seen in New York City where a study found that the absence to safe and private sanitation and hygiene services among women exacerbates menstrual stigma [23]. The reduced access to sanitation and hygiene services creates feelings of embarrassment and shame that “hinder women's ability to be comfortable during their periods” and attend to their personal daily activities [23]. Sebert Kuhlmann et al.'s (2019) study also explored the experiences of unhoused women in St. Louis, Missouri and concluded that the inability to afford hygiene products resulted in women engaging in various coping mechanisms, including using rags, tissues, toilet paper, children's diapers, or paper towels to manage menstruation [35]. Another study in Manhattan, New York, found that spatial bias exists in the distribution of public sanitation facilities, with higher quality public toilets facilities located in affluent neighborhoods and poorer quality toilet facilities available around unhoused communities [36]. This form of environmental injustice in the distribution of goods limits access to sanitation that is private, safe, and accessible among unhoused women managing menstruation [23]. Overall, the absence of basic WaSH services to maintain a certain appearance and hygiene practices reinforces a cycle of homelessness as seen in Los Angeles and in other cities. The prejudicial attitudes towards unhoused people based on their physical appearance lead to exclusionary policies and further stigmatization that impacts people's ability to exist in public spaces and exit homelessness [46].

In our study we also found temporal access to WaSH services in Los Angeles. Specifically, the evening was the most challenging time for people to access sanitation services that are both open and safe to use. While 14% of the study population reported that they openly defecate during the afternoon, 28% are forced to openly defecate at night. Business establishments typically close at 9:00 PM in the community, and most non-profit organizations at the time were not available 24-h, except for the ReFresh Spot, the People Concern, and the Union Rescue Mission shelter. As a result, accessing WaSH services is severely limited for an estimated 1,898 unhoused individuals living in the community of Skid Row at night [47]. In the morning and afternoon, participants reported utilizing public toilets in parks and libraries and toilets from non-profit organizations (e.g., shelters, soup kitchens, mobile showers, and religious organizations). These places tend to be free and open to the community until closure. However, while services may be more available during the morning and afternoon, participants reported long wait times, inconvenient hours, or out-of-service facilities. These factors discourage a person from maintaining hygiene practices, and forces them to resort to coping strategies, such as showering using buckets inside their tents, rinsing, and doing laundering in sinks of businesses and public toilets and throwing away their clothing rather than washing it.

### Inequities in WaSH access

This study findings suggests that there are community differences in the access to WaSH services that that unhoused people reported, and the Skid Row community is a service hub area, compared to downtown Los Angeles and the greater Los Angeles area. Participants who reported sleeping in locations outside Skid Row boundaries such as Santa Monica Beach, Hollywood, or South Los Angeles commuted by bus, metro, and or foot to access services (mainly shower and laundry facilities) in Skid Row. The commute from these neighbourhoods to Skid Row exceeds the JMP global standards for accessing drinking water and or other WaSH services of 30-min [3]. These participants also expressed that they commuted to Skid Row in the morning and afternoon to access services but left the area at night due to safety reasons. WaSH services outside of Skid Row boundaries are rarely available due to community opposition and criminalization of homelessness through city ordinances.

In Los Angeles, two major city ordinances exist that are heavily enforced: 1) the Los Angeles Municipal Code (LAMC) 41.18(d) that prevents people from sleeping in public areas between the hours of 6 AM to 9 PM [48] and 2) the LAMC 56.11 a city ordinance that limits unhoused people from having personal property exceeding the equivalent of a 60- gallon container [49]. The enforcement of these city ordinances overlap with street sweeps that sanitation workers conduct to remove encampments across Los Angeles. These sweeps disrupt WaSH service connections for unhoused communities that are displaced. Moreover, police enforcement criminalizes other coping behaviours, including public urination and open defecation, perpetrated due to inadequate access to WaSH services [50, 51]. The passage of such anti-homelessness laws in Los Angeles creates environments that reinforce a cycle of poverty and WaSH insecurity. It produces a system that punishes a vulnerable population for their existence and a criminal justice system that views them as pollution and a threat while actively diminishing an unhoused person’s ability to exist in public spaces [52, 53]. In Los Angeles, individuals are criminalized daily for their survival and coping mechanisms (e.g., sleeping in tents/vehicles and public urination/defecation), leading to infraction notices, misdemeanours, unpayable fines, and incarceration [50, 54]. These misdemeanours result in a criminal record that prevents people from qualifying for most housing services and employment, creating a cycle of sustained poverty [22, 33, 54–57]. The lack of publicly available WaSH services in Los Angeles serves as a form of oppression for a population that is often removed from public spaces to limit their visibility and potential disruptiveness [53, 58]. As essential WaSH services remain difficult for unhoused communities to access, reports of health outbreaks attributed to poor living environments and hygiene have become more pronounced in recent years [59, 60].

While unhoused people residing in Los Angeles in our study experienced WaSH insecurity, their experiences were not homogenous. WaSH insecurity is experienced differently among unhoused people, particularly for racially minoritized groups and people who sleep outside of Skid Row. Women are especially vulnerable and are forced to cope with limited access to sanitation and shower facilities on top of the economic burden of managing their menstrual cycle. Some participants mentioned that they experienced discrimination while waiting in line to use sanitation and body hygiene services. Specifically, Black and Latinx unhoused participants reported experiences of discrimination that prevented them from accessing shower services in shelter systems and restrooms in business and public establishments. Unhoused men also reported experiencing discrimination when accessing WaSH services more often than women, reducing their access to services that can meet their basic needs. Additionally, the trauma of being unhoused and being exposed to stressful WaSH environments can affect people differently. For women, openly defecating or showering inside their tents can pose a risk of physical violence or harassment. Additionally, in this study, six out of thirty-five female participants reported no longer having their menstrual cycle. Unhoused women who no longer have their menstrual cycle (a condition referred to as amenorrhea that affects one percent of the general population) may be due to trauma and stress-induced living on the streets [61]. Still, more measurements are needed to validate this finding. Of the 35 women that reported they continue to manage their menstrual cycle, 8 (or 22%) reported difficulty accessing feminine hygiene products. Generally, feminine hygiene products are expensive to purchase for the unhoused. Menstrual hygiene products are also not provided consistently in safety-net programs and shelter systems. The work of Kulhmann et al. (2019) in Missouri also reported this added barrier for low-income women. Kulhmann et al. states that the inability to afford high-cost products becomes an added burden for women, particularly when they cannot use federally funded programs (e.g., Women, Infants, and Children and Supplemental Nutrition Assistance Program) to purchase hygiene products [35].

### Impacts of WaSH insecurity

The barriers to maintaining good hygiene are numerous for unhoused people. For example, shower facilities are not always near participants, with some commuting long distances to access these services. Even when shower services are available in communities like Skid Row, it does not guarantees people access, as participants must sign up early or they will be part of a long waitlist process that can last all day. Additionally, shower access is subject to be inconsistent because of out of order or closed facilities. Overall, improving unhoused people’s ability to shower regularly could help decrease skin-related diseases, the leading cause for which unhoused people seek medical services [24, 62–65], among other health problems. In our study, sixty-four participants reported experiencing skin infections. While we did not collect information on the type of skin lesions and infections affecting participants, Leibler et al.’s (2017) study in Boston, Massachusetts found that unhoused people experience a higher prevalence of nasal colonization of staph compared to the general population. Leibler et al. also found 16 unhoused people with MRSA nasal colonization resulting from limited hygiene and crowded living conditions [24]. Overall, skin conditions are made worse by a lack of sanitation and poor hand and body hygiene practices, putting unhoused individuals at a higher risk of infection. Furthermore, access to laundry services is limited by affordability and availability. In our study, 48% of participants reported relying on non-profit organizations for laundry services which charge small fees for laundering or are limited by hours of operation. While 39% of participants reported using private laundromats, access was limited by affordability and proximity. In Los Angeles, the requirement of customer-only access to toilets and or based on appearance drastically reduces the well-being and capacity of unhoused people to maintain good sanitation practices, forcing people resort to openly defecating or using buckets inside their tents.

Other endemic poor health outcomes in unhoused communities are head and body lice, scabies, and secondary bacterial infections, all of which can be WaSH preventable diseases [63]. In this study, nine participants reported having body and head lice within a 30-day period. These numbers are much smaller than other studies, including Bonilla et al.'s (2014) study in San Francisco with 203 unhoused people, of whom ten people had head lice and 60 reported body lice. Lice infestation can affect unhoused residents as they do not have consistent and reliable access to clean changes of clothing or bathing facilities [63, 66–68]. In this study, we only found that a total of five participants had typhus, which is relatively more minor compared to Badiaga et al.'s (2012) study in Marseilles, France, which detected sixty-three people with antibodies against *Rickettsia typhi* [69]. This vector disease causes murine typhus. One of the reasons for these differences may be that more comprehensive testing is needed to measure the prevalence of this poor access to WaSH-related health outcomes.

Furthermore, dehydration and urinary tract infections were common health conditions reported by the sampled population. In this study, 34% of participants reported experiencing dehydration in the past 30 days from the time of interview. While the data collection took place in the two hottest months of the year (June and July) in 2019, heat exposure and lack of available drinking water can result in heat exhaustion. More than half of the sampled population reported consuming up to three (16 oz) bottles of water per day, which is less than the recommended 3.7 L (125 oz) and 2.7 L (67 oz) per day water intake for men and women in the US, respectively [64, 70]. In the community of Skid Row, there is a limited number of public water fountains available, and those that are in place are poorly maintained, reducing access to safe drinking water for this population. The limited access to public drinking water facilities increases the risk of dehydration, heat exhaustion, and urinary tract infections. DeMeyers, Warpinski, and Wutich's (2017) study in Arizona found that lack of vegetation, urban heat island effect, and lack of WaSH services are all factors that increased the risks of dehydration and heat exhaustion [22] for the unhoused. Lastly, we found that several people reported holding off from using the toilet, especially in the evening time when facilities are closed and inaccessible. These coping strategies can lead people to encounter health problems like kidney and vaginal infections. In our study, 32 participants reported urinary tract infections within 30 days of the interview, and 18 were women. Urinary tract infections can result when people delay using a toilet, and lack of adequate access to WaSH services can increase their risk of contracting infections [23, 71]. Women are also at higher risk of contracting kidney and vaginal infections. For example, Wenzel et al.'s (2001) study found that many unhoused women in Los Angeles County encounter gynaecological symptoms. However, it is important to also note that there may be other risk factors of urinary tract infections beyond the lack of WaSH services [73, 74]. In addition to WaSH services, better health care support systems are needed to address unhoused women's needs [72].

### Limitations

There are some limitations to this study that can inform future studies of the unhoused. First, this study only surveyed 263 participants, accounting for less than one percent of the County and City of Los Angeles's total unhoused population. A larger sample could improve statistical power for detecting effects. Second, the population is difficult to reach and access, so we used a mixture of snowball and convenience sampling to recruit participants. A random sampling technique to recruit participants would have made the results more generalizable. Additionally, the locations where we sampled some of the population may be attributed to location bias. We partnered with a WaSH non-profit organization on two occasions to provide us with a safe space to recruit participants, which may have led to oversampling the population who knew of and use the services provided by the non-profit. As a result, there may have been newly unhoused people who were unaware that these services existed, so they were not interviewed. This study may overestimate WaSH access among the unhoused as people were surveyed during the hottest two months of the year in Los Angeles (June and July). The reported WaSH insecurity experiences may have been different and or exacerbated during these heatwave months given that there is no green space in Skid Row and the community is an urban heat island. The type of questions we asked participants are based on self-reported WaSH access which may have resulted in recall bias. Participants may not have remembered all their daily habits within the timeframe given, specifically the WaSH related health outcomes they experienced over a 30-day period. At the same time, the health outcomes data that we collected may not only be directly linked to poor WaSH access. For example, constipation, urinary tract infections, and migraines have other causes beyond poor access to WaSH services. Since the unhoused population is mobile, there may have also been duplicate interviewees. If recognized, a survey was either omitted from the final analysis or used to validate their initial survey responses.

### Recommendations

More extensive mixed methods studies are needed to disentangle WaSH access among different cohorts to understand how poor access to WaSH services affects unhoused communities differently. Future research could integrate an intersectionality lens to consider the range of effects of WaSH insecurity on different groups among the unhoused to raise awareness on the inequity, marginalization, and discrimination at the individual and structural levels. While this study captures some of these vulnerabilities, it does not capture all the intersectional vulnerabilities experienced among unhoused communities. It is important to highlight the marginalized identities among the unhoused, including people who identify as transgender, undocumented immigrants, people who inject drugs, and adults over the age of 50 years. Examining the experiences of marginalized groups, not examined in this study, can help us understand and acknowledge the added vulnerabilities people experience in accessing services and exiting homelessness.

Future studies should consider how to measure network analysis of WaSH access. For example, identifying the best location to provide WaSH services in the different communities and characteristics of effective interventions could be identified to make WaSH services more accessible to this population. Additionally, studies should consider measuring the psychosocial health outcomes attributed to WaSH insecurity among unhoused communities. Measuring emotional distress is critical to capture in both scholarly research and policy implementation as unsafe and inadequate access to essential WaSH services can lead to emotional distress and exacerbate mental health diagnoses. Lastly, future studies should consider exploring the effects of WaSH insecurity on medication adherence in the Los Angeles unhoused population [75, 76].

There is a need to prioritize safe, dignified, affordable, sufficient, reliable, and continuous access to public and mobile WaSH services in vulnerable communities, including people experiencing homelessness [8]. To better understand and mitigate WaSH insecurity in the US and worldwide, we need to move beyond the lens of household WaSH insecurity and include mobile people, like those experiencing homelessness. Furthermore, in Los Angeles, a collaboration between service providers, policymakers, healthcare systems, and researchers is needed to develop inclusive and equitable solutions. Indeed, improving the way we address homelessness requires an integrative process. Service providers, particularly in the non-profit sector, play a vital role in this process as they work directly with the community and know their service needs.

The provision of housing with integrated services, including WaSH services, can lead to a more comprehensive response to the needs of unhoused communities. The findings from this study highlight that providing safe WaSH services at all times of the day is needed to meet the needs of unhoused communities in Skid Row and the surrounding areas. In Skid Row, the ReFresh Spot is an example of a successful model that works. Community members use the ReFresh Spot because the facilities are well maintained, have friendly staff, are clean, and available when needed. City officials could consider allocating money to WaSH infrastructure and facilities like the ReFresh Spot rather than installing temporary portable toilets and conducting encampment sweeps. In 2018, Los Angeles City officials spent 31 million dollars on street-clean ups [77]. These types of program interventions are not sustainable, humane, and do not target the root of the problem. Lastly, integrating the voices of unhoused residents at the decision-making tables could foster real change and improve these communities' health and living environments because they have the lived expertise and know what services are most valuable and needed.

Improving access to WaSH among the unhoused in Skid Row could also help the United States meet the Sustainable Development Goals (SDGs) 1, 2, 3, 5, and 6 on ending poverty and hunger, ensuring healthy lives, gender equality, and sustainable management of water and sanitation [78, 79]. Reduction in WaSH-related diarrheal disease can help individuals retain the nutrients they are consuming (SDG 2 and 5). Furthermore, increasing access to sanitation and hygiene services could help address gender inequalities and improve safe menstrual hygiene management for people who menstruate and are unhoused (SDG 6) [23, 80, 81].

To bring WaSH services to unhoused communities, whether be it through mobile or permanent WaSH facilities, or through Housing First programs, fiscal capital investment is needed to construct, operate, and maintain these services over the long term [17, 82]. In Los Angeles, the majority of existing WaSH services are provided by the non-profit sector. The non-profit sector relies on raising funds to construct, operate, and maintain WaSH services and these reoccurring costs can be difficult to sustain [82, 83]. In some instances, mobile non-profit services are forced to relocate their services as a result of encampment sweeps that disconnects unhoused community members from services they need and providers from the communities they need to serve.

## Conclusion

In this study, we found a lack of WaSH services for unhoused people in Skid Row, Los Angeles. Although high-income countries like the US report high rates of access to basic WaSH services, vulnerable and disadvantaged populations, including unhoused communities, experience WaSH insecurity daily. This study sheds light on the daily challenges and coping strategies of unhoused communities in Los Angeles where there is a lack of sufficient, safe, affordable, reliable, and continuously accessible WaSH services for this population. Access to safely managed sanitation services is most difficult and unreliable at night, as there are only a limited number of facilities open at night. Due to the inaccessibility of WaSH services, many unhoused people engage in different survival coping strategies. At the same time, the lack of basic WaSH services for this vulnerable population can result in a cycle of poverty, prolonged homelessness, deterioration of physical and mental wellbeing, and further stigmatization. There is a need for investment in WaSH infrastructures and the operation and maintenance of those services over the longer term to address WaSH insecurity experienced among unhoused communities in Los Angeles. This is crucial to meeting basic human rights, the SDGs, and reducing the spread of enteric and infectious diseases, including COVID-19.

## Supplementary Information

**Additional file 1. **Homelessness and WaSH Insecurity in Los Angeles Survey Instrument.

## Data Availability

The dataset used and/or analysed are available from the corresponding author on reasonable request. The survey instrument used in this study is provided as supplementary information accompanying this paper.
