# Supplementary material for: Water, Sanitation, and Hygiene (WaSH) insecurity in unhoused communities of Los Angeles, California
Source: Int J Equity Health. 2023 Jun 1;22:108. doi: 10.1186/s12939-023-01920-8 (PMC10233557; doi:10.1186/s12939-023-01920-8)
Supplement: Supplementary file 1 — Additional file 1. Homelessness and WaSH Insecurity in Los Angeles Survey Instrument. [file 12939_2023_1920_MOESM1_ESM.docx]

**Homelessness and Water Insecurity in Los Angeles**

**Interviewer Instructions**: Please read the following statement to participants

*My name is ___________ and we are from the Spatial Sciences Institute at USC. We were wondering if we could have a few minutes of your time to ask you a couple of questions. We are conducting a study that is trying to understand the ways you cope with water scarcity and how you access water for drinking, bathing, using a toilet, and laundry services. We are interested in learning about the challenges you face in finding these basic human needs, as we hope in the future to advocate to increase the access to these services in public areas in Los Angeles County.*

*Would you like to participate in this study? [If Yes] Before we begin, I want to remind you that your participation is based on a survey and that it is completely voluntary. We will not be collecting any identifiable information and everything in this survey will be kept* ***confidential****. If at any point in this survey there is a question or statement that you do not wish to answer or feel embarrassed to answer, please let me know and we will skip it and move on to the next question.*

Have you been interviewed by a USC student in the past week?

- Yes **→** **STOP** **interview**. Thank them for their time and continue with the next person
- No **→** Continue with Survey

Are you over the age of 18?

- Yes **→** Continue with Survey
- No **→** **STOP interview**. Participant does not qualify. Thank them for their time and move on to the next person

Do you consider yourself to be homeless or unstably housed?

- Yes **→** Continue with Survey
- No **→** **STOP interview**.

Today’s Date: ________________

Today’s Weather: _______________

Participant’s ID:

Interviewer:

Study Area:

- Skid Row
- Greater DTLA Area
- Freeway/Bridge

Survey Completed:

- Yes
- No

**Where do you usually stay/sleep?**

(Refer to street intersections/ or shelter)

[DROP PIN IN MAP]

First Street

Second Street

**OR** include name of Sleeping Location/Shelter: ______________________________

**Section I. Mobility and Financial Information**

*This first section contains statements about your living situation, financial support, and personal constraints accessing basic water resources. Our goal is to get an idea of how far you may have to walk to access services and your biggest needs and services of value to you. Please answer the statements to the best of your ability.*

1. How many months/years have you been homeless or unstably housed?

- ______ months
- ______ years
- Don’t know
- Prefer not to answer

1. In the past **30 days**, let me know if you have stayed in any of the following types of places, even for one night?  (Check all that apply)

- Tent
- Outdoors (not in a tent)
- Vehicle
- Own house, apt, hotel room
- Temporary hotel
- Rented room in someone's place
- With family, friend
- Shelter
- Hospital
- Jail or prison
- Residential treatment
- Group home
- Other: _________________
- Don't know
- Not applicable
- Prefer not to answer

1. What do you think are some of the main reasons or conditions that led you to you losing your home?

- Unemployment
- Divorce, Separation, or Break-Up
- Domestic Violence
- Sexual Abuse
- Conflict with Family/Friends
- Family/Spousal Death
- Physical Disability
- Mental Health Issues
- Drug or Alcohol Use
- Rising Housing Cost
- Eviction or Foreclosure
- Other: __________
- Prefer not to answer

1. In the past 30 days, **did you move** your tent/belongings to a different location (including across the street or down the block) and **how many times**?

- Yes, ______________ (number of moves) **🡪 Go to PARTS A and B**
- No/ Not applicable
- Don’t know/Remember
- Prefer not to answer

***If Q.4 is equal to NO, then skip to the next question.***

- 1. In the past **30 days**, how many of these moves were because of police, security guards, or other city/county officials?
- _______ (number of moves)
- Don’t Know/Remember
- Not Applicable
- Prefer not to answer
  1. In the past **30 days**, how many of these move were because of sanitation workers and street clean-ups?
- ________ (number of moves)
- Don’t Know/Remember
- Not Applicable
- Prefer not to answer

1. In the past 12 months, have you ever been **given a citation/ticket** and how many times?

- Yes, _____________ (times) 🡪 **Go to PART A-C**
- No
- Don’t Know/Remember
- Prefer not to Answer

***If Q.5 is equal to NO, then skip to the next question.***

- 1. Do you remember the amount of those citations/tickets?
- Yes, _______ ($ amount)
- No
- Don’t know/Remember
- Prefer not to Answer
  1. Was the citation/ticket for public urination?
- Yes
- No
- Don’t know/Remember
- Prefer not to Answer
  1. Did you had to go to jail for this citation and or marked as a sex offender?
- Yes
- No
- Don’t know/Remember
- Prefer not to Answer

1. Do you receive any public assistance and what is the **amount**? (DO NOT read options)

- Yes, Public Housing
- Yes, Food Stamps/Cal Fresh/SNAP: _________ ($ amount)
- Social Security: _________ ($ amount)
- Medicaid/Medi-Cal
- General Relief: _________ ($ amount)
- Section 8
- Other: ___________
- None
- Prefer not to Answer

1. Do you have difficulty accessing any of the following services? (Select all that apply)

- Restrooms (toilets)
- Showers
- Drinking Water
- Water Fountains
- Laundry Services
- Sinks for Handwashing
- Hygiene supplies (soap, toothpaste, razor, tampons/pads, toilet paper, etc.)
- Health supplies (needles, overdose kit, medicine, etc.)
- None of the Above – all these resources are easily available to me
- Prefer not to Answer

**Section II. Discrimination**

*This brief section contains statements about your perceived experiences in accessing services*

1. Have you ever felt discriminated in any way when trying to access any of these resources?

- Yes, when trying to use a **restroom**
- Yes, when trying to use a **shower**
- Yes, when trying to get **drinking water**
- Yes, when trying to **wash my clothes**
- Yes, when trying to **wash my hands**
- Yes, when trying to get **hygiene supplies**
- Yes, when trying to get **health/medical supplies**
- No, never
- Prefer not to answer

1. Have you ever felt discriminated because of the following reasons?

- Age
- Race
- Gender
- Sexual Orientation
- Disability Status
- Citizenship Status
- Religion
- Other/more detail: _______________________________
- None of the Above – never felt discriminated
- Prefer not to answer

**Section III. Personal Hygiene**

*The next series of questions are about* ***your*** ***personal hygiene****. Some of these questions could be embarrassing to answer. If at any point in this survey there is a question that you do not wish to answer, please let me know and we will skip that question or conclude the survey.*

**RESTROOM ACCESS:**

1. When you need to **urinate**, how easy or hard is it for you to find a toilet? (Mark an X)

| Very easy | Easy | Neither easy or hard | Hard | Very hard | Prefer not to answer |
| --- | --- | --- | --- | --- | --- |

1. When you need to **defecate**, how easy or hard is it for you to find a toilet? (Mark an X)

| Very easy | Easy | Neither easy or hard | Hard | Very hard | Prefer not to answer |
| --- | --- | --- | --- | --- | --- |

1. Upon waking up in the **MORNING**, where do you go to use a **RESTROOM**?

- Refresh Spot
- Shelters/Missions (include name/s): __________________________________________________________
- Public Restrooms (PIT Stop, libraries, parks, etc.)
- Streets, Sidewalk, or Doorway
- In Tent using a bucket or other equipment: ___________________________________________________
- Inside restaurants, stores, etc. (include name/s): _____________________________________________
- Other/more detail _______________________________________________________________________________
- Prefer not to answer

***If Q12. is equal to Shelters/Restrooms/Restaurants, then skip to the next question.***

- 1. What do you do to dispose of the waste? (DO **NOT** READ OPTIONS)
- Dispose in drainage
- Dispose in trash bin/can
- Dispose in bag
- Dispose in the street
- Other: ____________________________
- Prefer not to Answer

1. In the **MORNING**, **how** **many blocks** does it take you to **walk** to find a **RESTROOM**?

- 1-2 blocks
- 3-4 blocks
- 5-6 blocks
- Other: _________ (estimated number of blocks)
- Don’t Know/Remember
- Not Applicable
- Prefer not to answer

1. In the **MORNING**, **how long** does it take you to **walk** to find a **RESTROOM**?

- 1-2 minutes
- 3-5 minutes
- 6-10 minutes
- 11-15 minutes
- Other: ___________________ (estimated number of minutes)
- Don’t Know/Remember
- Not Applicable
- Prefer not to answer

1. In the **MORNING**, **how long** do you have to **wait** to use a **RESTROOM**?

- No wait
- 1-2 minutes
- 3-5 minutes
- 6-10 minutes
- 11-15 minutes
- Other: ___________________ (estimated number of minutes)
- Don’t Know/Remember
- Prefer not to answer

1. Why do you go to these places in the **MORNING,** rather than somewhere else?

- It’s free/affordable
- It’s open when I need it
- It’s not crowded/I don’t have to wait a long time
- It’s close to where I stay
- It’s clean
- I feel safe there
- It’s wheelchair accessible
- It’s child/pet friendly
- The staff/security guards are friendly and welcoming
- Other/more detail: ______________________________________________________________________
- Prefer not to answer

1. In the **AFTERNOON**, where do you go to use the **RESTROOM**?

- Refresh Spot
- Shelters/Missions (include name/s): __________________________________________________________
- Public Restrooms (PIT Stop, libraries, parks, etc.)
- Streets, Sidewalk, or Doorway
- In Tent using a bucket or other equipment: ___________________________________________________
- Inside restaurants, stores, etc. (include name/s): _____________________________________________
- The same place/s as in the morning time
- Other/more detail _______________________________________________________________________________
- Prefer not to answer

1. In the **AFTERNOON, how many blocks** does it take you to **walk** to find a **RESTROOM**?

- 1-2 blocks
- 3-4 blocks
- 5-6 blocks
- Other: __________ (estimated number of blocks)
- Same as the morning-time
- Don’t Know/Remember
- Not Applicable
- Prefer not to answer

1. In the **AFTERNOON**, **how long** does it take you to **walk** to find a **RESTROOM**?

- 1-2 minutes
- 3-5 minutes
- 6-10 minutes
- 11-15 minutes
- Other: ___________________ (estimated number of minutes)
- Same as the morning-time
- Don’t Know/Remember
- Not Applicable
- Prefer not to answer

1. In the **AFTERNOON**, **how long** do you have to **wait** to use a **RESTROOM**?

- No wait
- 1-2 minutes
- 3-5 minutes
- 6-10 minutes
- 11-15 minutes
- Other: ___________________ (estimated number of minutes)
- Same as the morning-time
- Don’t Know/Remember
- Prefer not to answer

1. Why do you go to these places in the **AFTERNOON,** rather than somewhere else?

- It’s free/affordable
- It’s open when I need it
- It’s not crowded/I don’t have to wait a long time
- It’s close to where I stay
- It’s clean
- I feel safe there
- It’s wheelchair accessible
- It’s child/pet friendly
- The staff/security guards are friendly and welcoming
- Other/more detail: ______________________________________________________________________
- Prefer not to answer

1. During the middle of the **NIGHT**, where do you go to use the **RESTROOM**?

- Refresh Spot
- Shelters/Missions (include name/s): __________________________________________________________
- Public Restrooms (PIT Stop, libraries, parks, etc.)
- Streets, Sidewalk, or Doorway
- In Tent using a bucket or other equipment: ___________________________________________________
- Inside restaurants, stores, etc. (include name/s): _____________________________________________
- The same place/s as in the morning time
- Other/more detail _______________________________________________________________________________
- Prefer not to answer

1. At **NIGHT, how many blocks** does it take you to **walk** to find a **RESTROOM**?

- 1-2 blocks
- 3-4 blocks
- 5-6 blocks
- Other: __________ (estimated number of blocks)
- Same as the morning-time
- Don’t Know/Remember
- Not Applicable
- Prefer not to answer

1. At **NIGHT**, **how long** does it take you to **walk** to find a **RESTROOM**?

- 1-2 minutes
- 3-5 minutes
- 6-10 minutes
- 11-15 minutes
- Other: ___________________ (estimated number of minutes)
- Same as the morning-time
- Don’t Know/Remember
- Not Applicable
- Prefer not to answer

1. At **NIGHT**, **how** **long** do you have to **wait** to use a **RESTROOM**?

- No wait
- 1-2 minutes
- 3-5 minutes
- 6-10 minutes
- 11-15 minutes
- Other: ___________________ (estimated number of minutes)
- Same as the morning-time
- Don’t Know/Remember
- Prefer not to answer

1. Why do you go to these places at **NIGHT,** rather than somewhere else?

- It’s free/affordable
- It’s open when I need it
- Other facilities are closed/out of service
- It’s not crowded/I don’t have to wait a long time
- It’s close to where I stay
- It’s clean
- I feel safe there
- It’s wheelchair accessible
- It’s child/pet friendly
- The staff/security guards are friendly and welcoming
- Other/more detail: ______________________________________________________________________
- Prefer not to answer

1. Have you ever been denied access to use a **BATHROOM** service, and if so, where did this happened?

- Yes, ___________________________________________________________________________(locations)
- No, I have never been denied access to use a bathroom
- Prefer not to answer

1. Why were you denied access to a **RESTROOM**?

- I have to be a customer
- It cost too much and could not afford it
- It was closed [out of service/outside hours of operation/in use/other
- I was turned away by staff, a guard, etc. because of my appearance
- It wasn’t child/pet-friendly
- Other/more detail: _________________________________________________
- None of the Above/ Not Applicable
- Prefer not to answer

1. Do you ever have to hold your pee at any time of the day because of a lack of public restrooms available and how often?

- Yes, all the time
- Yes, sometimes
- Yes, rarely
- No, never
- Don’t know/Remember
- Prefer not to answer

**SHOWER ACCESS:**

1. In the last week, how often have you changed your clothes?

- 1-2 times
- 3-4 times
- 5-6 times
- Everyday
- When I can wash my clothes
- Don’t Know/Remember
- Prefer not to Answer

1. How many times have you had a **BATH/SHOWER** in the week?

- 1-2 times
- 3-4 times
- 5-6 times
- Everyday
- Don’t Know/Remember
- Prefer not to Answer

1. Do you prefer to shower in the morning, day, or night-time?

- Morning
- Day/Afternoon
- Night
- Not applicable
- Prefer not to Answer

1. At that time of the day is it more difficult to use take a **SHOWER**?

- Morning
- Day/Afternoon
- Night
- None
- Prefer not to answer

1. What places do you visit regularly to **SHOWER**?

- Refresh Spot
- LAMP/People’s Concern
- Lava Mae/Other Mobile Showers
- Shelters/Missions (include name/s): __________________________________________________________
- Public Restrooms (PIT Stop, libraries, parks, etc.)
- In Tent (using a bucket or other equipment: __________________________________________________
- Inside restaurants, stores, etc. (include name/s): _____________________________________________
- Church/Religious organizations (include name/s): ___________________________________________
- Other/more detail _______________________________________________________________________________
- Not Applicable
- Prefer not to answer

1. How **many blocks** does it take you to **walk** to find a **SHOWER**?

- 1-2 blocks
- 3-4 blocks
- 5-6 blocks
- Other: ___________ (estimated number of blocks)
- Don’t Know/Remember
- Not Applicable
- Prefer not to answer

1. How **long** does it take you to **walk** to find a **SHOWER**?

- 1-2 minutes
- 3-5 minutes
- 6-10 minutes
- 11-15 minutes
- Other: ___________________ (estimated number of minutes)
- Don’t Know/Remember
- Not Applicable
- Prefer not to answer

1. How **long** do you have to **wait** to use a **SHOWER**?

- No wait
- 5-10 minutes
- 15-30 minutes
- 60-90 minutes
- Other: ___________________ (estimated number of minutes/hours, please specify)
- Don’t Know/Remember
- Not Applicable
- Prefer not to answer

1. Are these places available 24 hours or at what time do these places stop running/close for the day?

- Yes, available 24 hours
- No, _____________ (time)
- Don’t know/Remember
- Prefer not to answer

1. Why do you go **SHOWER** in this place/these places rather than somewhere else?

- It’s free/affordable
- It’s open when I need it
- It’s not crowded/I don’t have to wait a long time
- It’s close to where I stay
- It’s clean
- I feel safe there
- It’s wheelchair accessible
- It’s child/pet friendly
- The staff/security guards are friendly and welcoming
- Other/more detail: _____________________________________________________________________________
- Prefer not to answer

1. Have you ever been denied access to use a **SHOWER** service, and if so, where did this happened?

- Yes, ___________________________________________________________________________(locations)
- No, I have never been denied access to use a shower
- Prefer not to answer

1. Why were you denied access to a **SHOWER**?

- I have to be a customer
- It cost too much and could not afford it
- It was closed [out of service/outside hours of operation/in use/other
- I was turned away by staff, a guard, etc. because of my appearance
- It wasn’t child/pet-friendly
- Other/more detail: _________________________________________________
- None of the Above/ Not Applicable
- Prefer not to answer

1. Of the places you frequent to shower or use the toilet, do they provide the following? (Mark each box that applies)

| **Supply** | **Yes** | **No** | **Sometimes** | **Maybe** | **Prefer not to answer** |
| --- | --- | --- | --- | --- | --- |
| Toilet Paper |  |  |  |  |  |
| Soap |  |  |  |  |  |
| Hand Sanitizer |  |  |  |  |  |
| Seat Cover |  |  |  |  |  |
| Paper Towels/Air Dryer |  |  |  |  |  |
| Trash Can |  |  |  |  |  |
| Feminine Hygiene Products  **(Females only)** |  |  |  |  |  |
| Toilet that flushes |  |  |  |  |  |
| Functioning Sink |  |  |  |  |  |
| Doors with Working Locks |  |  |  |  |  |
| Well-Lit |  |  |  |  |  |
| Toothpaste/Toothbrush |  |  |  |  |  |
| Towel |  |  |  |  |  |
| **Other: ___________________** |  |  |  |  |  |

**DRINKING WATER ACCESS:**

1. What places do you visit regularly to get **DRINKING WATER** in the **MORNING?**

- Donations
- Public Water Fountains
- Fire Hydrant
- Supermarket/Liquor store (self-purchase)
- Restaurant, stores etc. (include name/s): _____________________________________________
- Shelters/Missions (include name/s): __________________________________________________
- Public Restrooms (PIT Stop, libraries, parks, etc.)
- Church/Religious organizations (include name/s): __________________________________
- Other/more detail _______________________________________________________________________
- Not Applicable
- Prefer not to answer

1. In the **MORNING,** how many blocks does it take you to **walk** to find **DRINKING WATER**?

- 1-2 blocks
- 3-4 blocks
- 5-6 blocks
- Other: __________ (estimated number of blocks)
- Don’t Know/Remember
- Not Applicable
- Prefer not to answer

1. In the **MORNING**, how long does it take you to **walk** to find **DRINKING WATER**?

- 1-2 minutes
- 3-5 minutes
- 6-10 minutes
- 11-15 minutes
- Other: ___________________ (estimated number of minutes)
- Don’t Know/Remember
- Not Applicable
- Prefer not to answer

1. What places do you visit regularly to get **DRINKING WATER** in the **NIGHT?**

- Donations
- Public Water Fountains
- Fire Hydrant
- Supermarket/Liquor store (self-purchase)
- Restaurant, stores etc. (include name/s): _____________________________________________
- Shelters/Missions (include name/s): __________________________________________________
- Public Restrooms (PIT Stop, libraries, parks, etc.)
- Church/Religious organizations (include name/s): __________________________________
- Other/more detail _______________________________________________________________________
- Not Applicable
- Prefer not to answer

1. During the **NIGHT,** how many blocks does it take you to **walk** to find **DRINKING WATER**?

- 1-2 blocks
- 3-4 blocks
- 5-6 blocks
- Other: __________ (estimated number of blocks)
- Same as the morning-time
- Don’t Know/Remember
- Not Applicable
- Prefer not to answer

1. During the **NIGHT**, how long does it take you to **walk** to find **DRINKING WATER**?

- 1-2 minutes
- 3-5 minutes
- 6-10 minutes
- 11-15 minutes
- Other: ___________________ (estimated number of minutes)
- Same as the morning-time
- Don’t Know/Remember
- Not Applicable
- Prefer not to answer

1. Why do you go to these places to get **DRINKING WATER**, rather than somewhere else?

- It’s free/affordable
- It’s available when I need it
- It’s not crowded/I don’t have to wait a long time
- It’s close to where I stay
- The water is clean
- I feel safe there
- It’s wheelchair accessible
- It’s child/pet friendly
- The staff/security guards are friendly and welcoming
- Other/more detail: ______________________________________________________________________
- Prefer not to answer

1. What is your **MAIN** source of drinking water?

- Bottled water from shelters/missions
- Bottled water from street donations
- River/Creek
- Water Fountains (from parks and libraries)
- Fire Hydrant
- Neighborhood Sprinkler/Water hose
- Free Water from Restaurants
- Other: _____________________
- Not Applicable
- Prefer not to answer

1. Have you ever been denied access to **DRINKING WATER**, and if so, where did this happened?

- Yes, ___________________________________________________________________________(locations)
- No, I have never been denied to drinking water
- Prefer not to answer

1. Why were you denied access to **DRINKING WATER** there?

- I have to be a customer
- It cost too much and could not afford it
- It was closed [out of service/outside hours of operation/in use/other
- I was turned away by staff, a guard, etc. because of my appearance
- It wasn’t child/pet-friendly
- Other/more detail: _________________________________________________
- None of the Above/ Not Applicable
- Prefer not to answer

1. Do you think you drink sufficient water throughout the day?

- Yes
- No
- Other: _______________________
- Prefer not to answer

1. On a daily basis, on average, how much drinking water do you **actually consume**?

- 0-3 bottles
- 4-6 bottles
- Greater than 6 bottles
- Don’t Know
- Prefer not to answer

**LAUNDRY SERVICES**

1. What places do you visit regularly to do **LAUNDRY**?

- Refresh Spot
- LAMP/People’s Concern
- Public Sink (PIT Stop, parks, libraries, etc.)
- Sinks in restaurants, stores, etc. (include name/s): __________________________________
- Shelters/Missions; include name/s: ___________________________________________________
- Private Laundromat (self-pay); include name/s: _____________________________________
- Other/more detail_______________________________________________________________________
- Prefer not to answer

1. Why do you go to **WASH YOUR CLOTHES** in this place/these places?

- It’s free/affordable
- It’s open when I need it
- It’s not crowded/I don’t have to wait a long time
- It’s close to where I stay
- It’s clean
- I feel safe there
- It’s wheelchair accessible
- It’s child/pet friendly
- The staff/security guards are friendly and welcoming
- Other/more detail: ______________________________________________________________________
- Prefer not to answer

1. How often do you **WASH YOUR CLOTHES**? (Mark an X)

| Everyday | Twice a Week | Once a Week | One a Month | None | Other | Prefer not to Answer |
| --- | --- | --- | --- | --- | --- | --- |

1. Have you ever been denied access to **WASH YOUR CLOTHES**, and if so, where did this happened?

- Yes, ___________________________________________________________________________(locations)
- No, I have never been denied to laundry services
- Prefer not to answer

1. Why were you denied access to **WASH YOUR CLOTHES** there?

- It cost too much and could not afford it
- It was closed [out of service/outside hours of operation]
- I was turned away by staff, a guard, etc. because of my appearance
- It wasn’t child/pet-friendly
- Other/more detail: _________________________________________________
- None of the Above/ Not Applicable
- Prefer not to answer

**HANDWASHING / SINKS**

1. How often do you wash your hands? (Check box that applies for each category)

| **Situation** | **Soap or Sanitizer** | **Always** | **Often** | **Sometimes** | **Rarely** | **Never** | **Prefer not to answer** |
| --- | --- | --- | --- | --- | --- | --- | --- |
| Before Preparing Food |  |  |  |  |  |  |  |
| Before Eating Food |  |  |  |  |  |  |  |
| After Using The Toilet |  |  |  |  |  |  |  |

1. Where do you go to **WASH YOUR HANDS**, when possible?

- Refresh Spot
- LAMP/People’s Concern
- Public Sink (PIT Stop, parks, libraries, etc.)
- Sinks in restaurants, stores, etc. (include name/s): __________________________________
- Shelters/Missions; include name/s: ___________________________________________________
- Church/Religious Organizations (include name/s): __________________________________
- Other/more detail_______________________________________________________________________
- Prefer not to answer

1. Why do you go to these places to **WASH YOUR HANDS,** rather than somewhere else?

- It’s free/affordable
- It’s open when I need it
- It’s not crowded/I don’t have to wait a long time
- It’s close to where I stay
- It’s clean
- I feel safe there
- It’s wheelchair accessible
- It’s child/pet friendly
- The staff/security guards are friendly and welcoming
- Other/more detail: ______________________________________________________________________
- Prefer not to answer

**HYGIENE SUPPLIES**

1. What **HYGIENE SUPPLIES** do you need the most but are **difficult** to obtain?

- Supplies needed: ________________________________________________________________________
- None, I can easily access everything I need
- Don’t know/Remember
- Prefer not to answer

1. Where do you obtain **HYGIENE SUPPLIES** when possible?

- Shelters/Missions; include name/s: ___________________________________________________
- Church/Religious Organizations (include name/s): __________________________________
- Self-bought in stores/pharmacies
- Non-profits (include name/s): _________________________________________________________
- Clinics (include name/s): _______________________________________________________________
- Other/more detail_______________________________________________________________________
- Prefer not to answer

**Section IV. Demographics**

*In this section, you will be asked* ***basic demographic*** *questions. If at any point in this section there is a question that you do not wish to answer, please let me know and we will skip the question.*

1. What is your age? ______________ (Write actual age)

- Prefer not to answer

1. What is your gender identity?

- Female
- Male
- Transgender female
- Transgender male
- Do not identify with any
- Other: ___________________________
- Prefer not to answer

1. What is your sexual orientation?

- Heterosexual
- Bisexual
- Homosexual
- Asexual
- Queer
- Not Sure/Questioning
- Other: ____________________________
- Prefer not to answer

1. Are you a U.S. citizen/permanent resident?

*If you do not feel comfortable answering them, we would skip these questions.*

- Yes
- No
- Prefer not to answer

1. What is your nationality? _________________________

- Prefer not to answer

1. What race/ethnicity do you identify yourself with?

- White/Caucasian
- Black/African American
- Latino/Hispanic
- Asian
- American Indian/Alaska Native
- Native Hawaiian/Pacific Islander
- Other _____________________________
- Prefer not to answer

1. What is your preferred language? ____________________________

- Prefer not to Answer

1. What is your current marital status?

- Single
- Married
- Separated
- Divorced
- Widowed
- Prefer not to answer

1. What is your highest level of education completed?

- No Education
- 8^th^ Grade or Less
- Some High School, Did Not Graduate
- High school diploma or GED
- Technical or vocational school
- Some college
- 4-Year college degree (Undergraduate)
- More than 4-year college graduate degree (Master’s, Doctoral, M.D. degree)
- Other _______
- Prefer not to Answer

1. Are you currently employed?

- Yes 🡪 **Go to PART A**
- No 🡪 **Go to PART B**
- Prefer not to answer

***If “YES,” complete the following question***

- 1. What is your current estimated **monthly** income? (Refer back to Q.6 Public Assistance)
- Answer: _____________________________ ($ monthly income)
- Prefer not to answer

***If “NO” complete the following question***

- 1. What is your current status?
- Retired
- Disabled
- Other___________
- Prefer not to answer

1. Have you ever served in the military?

- Yes 🡪 **Go to PART A**
- No
- Don’t Know
- Prefer not to answer

***If “NO,” skip and move on to the next question***

1. What is your status?

- Active
- Reserve
- Honorable Discharge
- Dishonorable Discharge
- General Discharge
- Other Than Honorable Discharge
- Retired/Disabled
- Prefer not to answer

**Section V. Health**

*The next questions are about your health conditions and access to health services. We understand that these questions could be embarrassing.  If at any point in this survey there is a question or statement that you do not wish to answer, please let me know and we will go on to the next question.*

1. In the past **12 months**, have you visited the Emergency Department and how many times?

- Yes: ______________________________ (times in the past year AND **reason** for visit)
- No
- Prefer not to Answer

1. How often do you access health care services?

- Not Often
- Rarely
- Sometimes
- All the time
- Do not access health services
- Other: __________________________________
- Prefer not to answer

1. What is your **biggest** health concern at the moment? __________________________
2. In the past **MONTH** (30 days), have you ever experienced or been diagnosed or any of the following health conditions? (Check all that applies)

- Dehydration
- Migraines/Headache
- Difficulty having bowel movement
- Skin infections (including staph, cellulitis)
- Urinary tract infections
- Head and or body lice
- Diarrhea/Loose stool
- Scabies
- Fungus
- Typhus
- Hepatitis A
- Other: __________________________________
- None of the above
- Prefer not to answer

1. In the past **YEAR** (12 months) have you ever experienced or been diagnosed with any of the following health conditions? (Check all that applies)

- Dehydration
- Migraines/Headache
- Difficulty having bowel movement
- Skin infections (including staph, cellulitis)
- Urinary tract infections
- Head and or body lice
- Diarrhea/Loose stool
- Scabies
- Fungus
- Typhus
- Hepatitis A
- Other: _________________________________
- None of the above
- Prefer not to answer

1. In the past **30 days,** how often have you had a bowel movement (able to poo)?

- 2 or more times a day
- Once a day
- 2 or more times a week
- Once a week
- 1-3 times a month
- Never
- Don’t know/Remember
- Not applicable
- Prefer not to answer

1. Have you ever received the **Hepatitis A** vaccine (it is a vaccine usually given to children and consist of 2 doses, administered 6 months apart)?

- Yes
- No
- Don’t know/Remember
- Prefer not to answer

1. In the past **MONTH** (30 days), have you ever stopped taking any prescribed medications because of the inability to cope with side effects (such as diarrhea, frequent urination, etc.) and difficulty accessing restrooms and other water services?

- Yes
- No
- Not applicable, not taking any medications
- Prefer not to answer

1. In the past **MONTH** (30 days), did you stopped you stopped taking any **psychiatric** medications because of the inability to cope with side effects?

- Yes
- No
- Not applicable, not taking any medications
- Prefer not to answer

1. In the past **30 days** have you taken hormonal therapy?

- Yes**🡪 GO TO PART A**
- No
- Prefer not to answer

***If “YES” answer the following question***

1. Did you had access to a restroom and proper hygiene services when taking hormonal therapy?

- Yes, how so: ____________________________
- No
- Not Applicable
- Prefer not to Answer

1. **FOR FEMALE STUDY PARTICIPANTS.** Do you still experience your menstrual cycle?

- Yes **🡪 Answer Questions A-C**
- No
- Not Applicable, Male Participant
- Prefer not to Answer

***If “NO” skip the following questions***

1. Do you have concerns or difficulties regarding hygiene during your periods? If so, what are your concerns?

- Yes, Answer: ___________________________________
- No
- Prefer not to Answer

1. Do you ever have issues acquiring feminine hygiene products? (For example: tampons, pads, wipes, etc.)?

- Yes, Answer: ___________________________________
- No
- Prefer not to Answer

1. Do you take birth control?

- Yes, which kind: ___________________________________
- No
- Prefer not to Answer

1. In the past **30 days**, where have you obtained your meals and what kind of foods did they served/did you purchased?

- Market (self-bought) ____________________________________________________________________
- Sit down Restaurant ____________________________________________________________________
- Fast-food Restaurant ____________________________________________________________________
- Mission/Soup kitchen___________________________________________________________________

1. On **average**, do you sleep in the day or night and how many hours of sleep?

- Morning, __________hours
- Night, _____________ hours
- Prefer not to answer

1. Have you ever been arrested/incarcerated?

- Yes, _____________________________________ (number of times AND reason)
- No, never
- Prefer not to answer

1. Do you have solid waste piles near your living area?

- Yes
- No, never
- Prefer not to answer

1. How frequently is trash collected in the area?

- Everyday
- Once a week
- Twice a week
- Rarely/Not Enough
- Other: ______________
- Prefer not to answer

**Section VI. Other Information**

1. Is there anything else that you would like to share with us?

Answer: ____________________________________________________________________________________________________________________________
